# The C Ring of the F1Fo ATP Synthase Forms the Mitochondrial Permeability Transition Pore: A Critical Appraisal

**DOI:** 10.3389/fonc.2014.00234

**Published:** 2014-08-25

**Authors:** Andrew P. Halestrap

**Affiliations:** ^1^School of Biochemistry and Bristol CardioVascular, University of Bristol, Bristol, UK

**Keywords:** mitochondrial permeability transition pore, necrotic cell death, F1Fo-ATPase, proteoliposomes, commentary

The mitochondrial permeability transition pore (MPTP) is a non-specific pore in the inner mitochondrial membrane (IMM) whose opening is triggered by high matrix [Ca^2+^] to which it is sensitized by [Pi] and oxidative stress. MPTP opening plays a critical role in necrotic cell death such as in cardiac ischemia/reperfusion (I/R) injury and the action of cytotoxic drugs. Indeed MPTP inhibition with cyclosporine A (CsA) protects tissues from I/R injury (1). Matrix cyclophilin D (CyP-D), the target of CsA, facilitates MPTP opening but the identity of the pore-forming proteins (to which CyP-D binds) remains unresolved (2–4). Extensive evidence supports the adenine nucleotide translocase (ANT) being the site of inhibition by adenine nucleotides and bongkrekic acid (BKA – an ANT ligand) and activation by carboxyatractyloside (CAT – another ANT ligand) and oxidative stress (5). Furthermore, the ANT binds CyP-D and, when reconstituted into proteoliposomes, it produces Ca^2+^-activated pores similar to the MPTP (6, 7). In addition, liver mitochondria lacking ANT1 and ANT2 exhibit MPTP opening that is insensitive to adenine nucleotides, CAT and BKA, and requires higher [Ca^2+^] than control mitochondria (8). However, since pore opening can still be observed, other IMM proteins must be able to form the MPTP. One candidate is the mitochondrial phosphate carrier (PiC), which binds CyP-D, more so following oxidative stress, and may be the locus of MPTP activation by Pi (9, 10). However, neither partial knockdown nor over-expression of PiC in cell lines affected MPTP opening (10, 11), although MPTP opening in heart mitochondria from mice with cardiac-specific PiC knockout were less calcium sensitive (12). Overall, the available data suggest that the ANT and PiC play roles in MPTP opening but that another IMM protein must also be involved. Several recent papers suggest that this may be the F1Fo ATP synthase.

CyP-D was shown to bind to the F1Fo ATP synthase and modulate its hydrolytic activity (13, 14) and in 2013, the laboratories of both Bernardi (15) and Pinton (16) presented data that implicated the ATP synthase in MPTP formation. Bernardi and colleagues (15) detected Ca^2+^-activated channels, similar to the MPTP, in phospholipid bilayers containing reconstituted dimers of mammalian F1Fo ATP synthase. Similar channel activity was demonstrated in yeast mitochondria and this was strongly attenuated in mutants lacking the ε and γ subunits needed for ATP synthase dimer formation (17). However, high levels (0.3 mM) of Ca^2+^ were required for channel opening which, unlike MPTP opening, also required Bz-423. No data were presented on the effects of oxidative stress, CsA, or recombinant CyP-D. Furthermore, Pinton and colleagues (18) pointed out that Bernardi’s laboratory had previously demonstrated MPTP opening in ρ^0^ cells, which lack the mitochondrial DNA encoding the α and A6L subunits of the ATP synthase. In addition, the ATPase inhibitor protein F1, which promotes ATP synthase dimerization, attenuates rather than promotes MPTP opening, and enhances cell survival under ischemic conditions (19). Rather, Pinton and colleagues (16) implicated the c-subunits of the Fo ATPase in MPTP formation, showing that their knockdown reduced MPTP opening in response to ionomycin or hydrogen peroxide and their over-expression enhanced opening. The c-subunits form a ring structure in the IMM, and so represent an attractive candidate for forming the MPTP, but direct evidence for this was not provided. However, the paper of Alavian et al. (20) claims to do this.

Alavian et al. (20) confirmed the observations of Bonora et al. (16), but they also reconstituted the purified c-subunit into proteoliposomes and demonstrated channel activity. Most channels conducted at ~100-pS but a few did so at 1.5–2 nS, similar to the MPTP conductance (21). However, the channels were insensitive to Ca^2+^ and CsA and were only inhibited by much higher concentrations of ATP and ADP than required to inhibit MPTP opening. The authors proposed that other F1Fo ATP synthase components are needed for MPTP regulation, which they investigated using purified monomeric F1Fo ATP synthase reconstituted into proteoliposomes. Some infrequent channel activity was observed, which was increased by addition of CyP-D and further by 100 μM Ca^2+^. These effects were prevented by 5 μM CsA, a concentration much higher than the K_i_ for CyP-D (2 nM). Channels sensitive to both Ca^2+^ and CsA could also be detected in patches from sub-mitochondrial vesicles (SMVs) enriched in F1Fo ATP synthase or IMMs and these were absent when OSCP and β subunits of the ATP synthase and bound CyP-D were removed by urea treatment, while adding back purified β-subunit to reconstituted c-subunits largely abolished channel activity. However, the authors did not address whether the IMM and SMV preparations also contained ANT and PiC, which is very likely. Rather, they concluded that the sites through which Ca^2+^, ADP, and CyP-D (and thus CsA) modulate channel activity are on the F1 domain of the ATP synthase whose association with the c-subunit ring may loosen upon Ca^2+^ and CyP-D binding. This might cause expansion of the ring converting it into a high conductance channel and, using fluorescent probes, they presented evidence consistent with the c-subunits moving apart during MPTP opening. Furthermore, channel activity was greatly enhanced when glycine residues in the c-subunit transmembrane domains were replaced with valines, thus moving the packed helices further apart (20). However, interpretation of these data is difficult because the expressed c-subunits ran at 15 kDa on SDS-PAGE and not 7.6 kDa, the size of the mature c subunit, suggesting that the mitochondrial targeting sequence had not been removed. Indeed several studies in this paper and that of Bonora et al. (16) showed expression of 15 kDa unprocessed protein rather than the true c subunit.

In summary, when the evidence for an involvement of the F1Fo ATP synthase and more specifically its c-subunit in MPTP formation is reviewed critically, it is legitimate to conclude that it is no better than that for the involvement of the ANT and PiC. Perhaps the truth lies in a synthesis, and that an interaction between the ANT, PiC, and F1Fo ATP synthase in the ATP synthasome (22) is critical for MPTP formation, as we (2) and subsequently others (4, 18) have concluded. A scheme illustrating how the different components may interact is presented in Figure 1.

**Figure 1:**
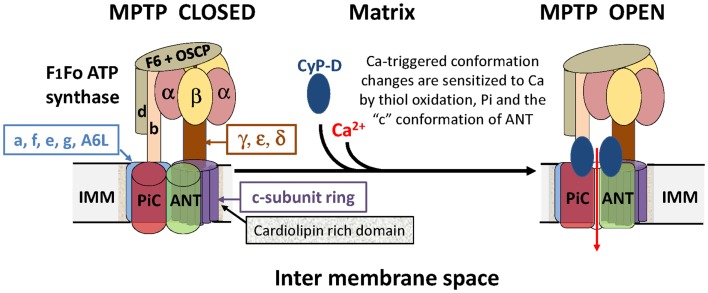
**A hypothetical model of the MPTP that proposes an interaction between the ANT, PiC, and F1Fo ATP synthase in the ATP synthasome**. It is proposed that the MPTP forms at the interface between interacting domains of the PiC, ANT, and Fo ATP synthase following calcium triggered conformational changes facilitated by the PPIase activity of CyP-D. Note that potential regulatory interactions of the MPTP with outer membrane components are not shown.

## Conflict of Interest Statement

The author declares that the research was conducted in the absence of any commercial or financial relationships that could be construed as a potential conflict of interest.
